# Development of novel *N*-methyl and *N*-allyl-substituted oxazolomorphinans and their interaction with opioid receptors

**DOI:** 10.1186/1471-2210-11-S2-A13

**Published:** 2011-09-05

**Authors:** Attila Sipos, Levente Girán, Sándor Berényi, Sándor Antus, Helmut Schmidhammer, Mariana Spetea

**Affiliations:** 1 Department of Organic Chemistry, University of Debrecen, 4010 Debrecen, Hungary; 2 Department of Pharmaceutical Chemistry, Institute of Pharmacy, and Center for Molecular Biosciences, University of Innsbruck, 6020 Innsbruck, Austria

## Background

The need for opioid analgesics with reduced undesirable side-effects has initiated a vast amount of scientific efforts, which have led to a number of new opioid ligands and significant expansion of knowledge in opioid pharmacology. The development of morphinans anellated with heterocycles gave rise to several potential therapeutic agents and useful pharmacological tools.

## Methods

The chemistry involved the design and synthesis of two sets of oxazolomorphinans having the new heteroring anellated to the A-ring of the morphinan backbone. Binding affinities of the newly synthesized compounds at opioid receptors were determined by *in vitro* competition binding assays using rat brain (μ, δ) and guinea pig brain (κ) membranes and employing [^3^H]DAMGO (μ), [^3^H][Ile^5,6^]deltorphin II (δ) and [^3^H]U-69,593 (κ) as specific opioid radioligands. The *in vitro* pharmacological activities were established using [^35^S]GTPγS functional assays in membranes from Chinese hamster ovary (CHO) cells expressing human opioid receptors.

## Results

Binding studies on the newly synthesized *N*-methyl and *N*-allyl derivatives to opioid receptors revealed remarkable results for three compounds: the amino-substituted *N*-methyloxazolomorphinan showed high affinity and selectivity to the μ opioid receptor, while two *N*-allyloxazolomorphinans were found to interact with high affinity with μ and κ sites and moderate binding towards δ receptors. In ligand-stimulated [^35^S]GTPγS binding studies, the *N*-methyl congener acted as a potent and full agonist at the μ receptor. The two *N*-allyl derivatives showed antagonistic effects at μ and κ receptors.

## Conclusions

The design and synthesis of novel oxazolomorphinans led to an interesting alteration in opioid activity by influencing the biological and pharmacological profile of these compounds interacting with μ, δ and κ opioid receptors.

